# Microarray Analysis of Visceral Adipose Tissue in Obese Women Reveals Common Crossroads Among Inflammation, Metabolism, Addictive Behaviors, and Cancer: AKT3 and MAPK1 Cross Point in Obesity

**DOI:** 10.1155/2024/4541071

**Published:** 2024-10-24

**Authors:** Rolando Martínez-Romero, Susana Aideé González-Chávez, Victor Roberto Urías-Rubí, Víctor Manuel Gómez-Moreno, Manlio Favio Blanco-Cantero, Héctor Mario Bernal-Velázquez, Arturo Luévano-González, César Pacheco-Tena

**Affiliations:** ^1^Laboratorio PABIOM, Facultad de Medicina y Ciencias Biomédicas, Universidad Autónoma de Chihuahua, Chihuahua, Mexico; ^2^Facultad de Medicina y Ciencias Biomédicas, Universidad Autónoma de Chihuahua, Chihuahua, Mexico; ^3^Instituto de Enseñanza en Salud de México, Ciudad de México, Mexico; ^4^Hospital Star Médica, Chihuahua, Mexico

**Keywords:** adipocyte, MAPK, microarray, PI3K/AKT/mTOR

## Abstract

**Background:** Visceral adipose tissue (VAT) abnormalities are directly associated with obesity-associated disorders. The underlying mechanisms that confer increased pathological risk to VAT in obesity have not been fully described.

**Methods:** A case-control study was conducted that included 10 women with obesity (36.80 ± 7.39 years, BMI ≥ 30 kg/m^2^) and 10 women of normal weight (32.70 ± 9.45 years, BMI < 24.9 kg/m^2^). RNA was extracted from greater omentum biopsies, and, using a DNA microarray, differential transcriptomic expression of VAT in women with obesity was evaluated taking as a reference that of women with normal weight. The differentially expressed genes (DEGs) were classified into functional biological processes and signaling pathways; moreover, the protein–protein interaction (PPI) networks were integrated for a deeper analysis of the pathways and genes involved in the central obesity-associated disorders. The expression of TNF-*α*, MAPK, and AKT proteins was also quantified in VAT.

**Results:** The VAT of women with obesity had 3808 DEGs, mainly associated with the cellular process of inflammation and carbohydrates and lipid metabolism. Overexpressed genes were associated with inflammatory, metabolic, hormonal, neuroendocrine, carcinogenic, and infectious pathways. Cellular processes related to addictive behaviors were notable. MAPK and PI3K-AKT pathways were overexpressed, and Mapk1 and Akt3 genes were common crossing points among obesity-associated disorders' pathways. The increased expression of MAPK, AKT, and TNF proteins was confirmed in the VAT of women with obesity.

**Conclusion:** VAT confers a complex and blended pathogenic transcriptomic profile in obese patients, where abnormal processes are mainly controlled by activating intracellular signaling pathways that exhibit a high degree of redundancy. Identifying shared cross points between those pathways could allow specific targeting treatments to exert a widespread effect over multiple pathogenic processes.

## 1. Introduction

Obesity is a complex multifactorial disease that has increased considerably in the last two decades; nearly a third of the world's population is now considered overweight or obese. Obesity affects several physiologic conditions and increases the risk for other disorders such as diabetes mellitus, cancer, cardiovascular disease, and a diversity of inflammatory conditions. Its multifactorial etiology includes genetic, environmental, socioeconomic, and behavioral or psychological factors [1].

Adipose tissue (AT) includes a diversity of cell strains; although it is mainly composed of adipocytes, it also includes inflammatory and immune cells, like macrophages and T-cells; in combination, they exert several physiologic and pathogenic roles. AT is considered a complex secretory organ with a wide array of roles in metabolism; it is known to play a central role in the pathogenesis of obesity. Adipocytes can modulate energy expenditure, hunger, insulin sensitivity, bone metabolism, reproductive and endocrine functions, inflammation, immunity, and energy reserve [2]. White AT is classified into visceral (VAT) and subcutaneous (SAT), whose differences lie in their metabolic and secretory profiles [3]. Increased visceral adiposity is more closely correlated than the body mass index (BMI) to predict the incidence of obesity-associated disorders.

Recent studies have shown significant differences between the VAT and the SAT. A high ratio of VAT to SAT has been linked to several morbid conditions [4–7], suggesting different physiological features and attributing further pathological capabilities to VAT. However, the precise molecular mechanisms that confer increased pathological risk to VAT in obesity have not been fully described. There is a certain degree of heterogeneity in the findings among existing studies, probably due to the influence of genetic and environmental factors on the obesity population.

Omics sciences, including transcriptomic, are reforming our understanding of the mechanisms explaining the complex biology behind obesity [8]. Transcriptomic profiles of VAT and SAT in obesity have been studied for more than 15 years [9–13], and several markers and signaling pathways have been described [14]; however, these efforts have not yet been sufficient to thoroughly understand obesity and its complications and stop the growth of this global epidemic, including the Mexican population, which has obesity rates increased by 42.2% between 2000 and 2018 [15]. Additionally, the notable developments in omics sciences in recent years, especially in bioinformatics capabilities, continue to search for crucial markers of this disease that can be used in strategies for its prevention and treatment. Therefore, the present study aimed to analyze the differential expression of VAT genes in Mexican women with morbid obesity compared to patients without obesity, using DNA microarrays.

## 2. Methods

### 2.1. Study Design

A case-control study was conducted to describe the differential transcriptomic expression of the VAT of patients with obesity from Chihuahua, Mexico, recruited from March to June 2022.

### 2.2. Participants

The cases included ten women with obesity according to the WHO criteria (BMI ≥ 30 kg/m^2^) of 18–50 years old who underwent elective gastric sleeve surgery and voluntarily agreed to participate in the study by signing informed consent. Those patients whose VAT samples were not suitable for genetic analysis were excluded. All patients were Mexican mestizos, a mixture of American indigenous backgrounds with Caucasian (mostly Spaniards) in different proportions.

The control group included ten women with BMI < 24.9 kg/m^2^ of 18–50 years old who underwent elective gynecological surgery, nine by laparoscopy (two endometriosis, one ovarian cyst, one salpingectomy, and five hysterectomies for uterine myomatosis) and two cesarean sections; the patients voluntarily agreed to participate in the study by signing informed consent. Patients were included when, after clinical assessment, they demonstrated the absence of chronic diseases, including cancer, thyroid disorders, diabetes, and hypertension. Pregnant patients, those treated with drugs that can alter metabolism and compromise body weight, and those whose VAT samples were unsuitable for genetic analysis were excluded.

The sample size was calculated according to what was described by Jung and Young [16] for microarray studies, considering 35,764 genes in the microarray, a sufficient number of 1 false positive, a statistical power of 80%, and a standard deviation (SD) of 70%. We obtained a sample size of 10 individuals per group.

### 2.3. Variables

Anthropometric variables were determined for both cases and controls, including height (cm), weight (kg), and BMI (kg/m^2^). In addition, for the group of patients with obesity, the % fat, body fat (kg), lean weight, visceral fat scale, % water, water weight (kg), muscle weight (kg), basal metabolic rate (Kcal), metabolic age, and daily calorie intake were also determined using the TANITA BF-350 bioelectrical impedance scale. Detailed anthropometrical measurements in the control group were unavailable since the control group patients were included from external clinics (mostly gynecological ones).

Differential transcriptome expression was determined using a DNA microarray containing 35,764 genes from the human genome by comparing the expression of RNAs from patients with obesity and those from patients without obesity. In addition, bioinformatic analysis was done to associate DEGs with signaling pathways and cellular processes.

The adipocyte size (*μ*M) and the expression of proteins of interest (optical density (OD)) were determined by light microscopy and compared between the VAT biopsies of both groups.

### 2.4. Sampling

The VAT biopsies were taken by bariatric surgeons and gynecologists from patients with and without obesity, respectively, during gastric sleeve or gynecological surgeries. VAT was obtained from the greater omentum. In brief, after the administration of general anesthesia, incisions from 1 to 3 cm were made, and with the use of the forceps of the laparoscopic kit, a VAT sample of approximately 2 cm^2^ was taken. The biopsy was divided into two and immediately preserved, one in liquid nitrogen and the other in 10% buffered formalin. The samples in liquid nitrogen were subsequently stored at −80°C until genetic analysis.

### 2.5. DNA Microarray and Bioinformatic Analysis

RNA was purified from visceral fat using the RNeasy Lipid Tissue Mini Kit (Qiagen, Hilden, Germany) extraction kit, according to the manufacturer's protocol. Integrity and quantity were verified in the Qubit4 fluorometer (Thermo Fisher Scientific, Waltham, MA, USA). Pools of RNA were made with equimolar amounts of RNA from each patient.

The DNA microarrays were performed at the Institute of Cellular Physiology, Autonomous University of Mexico (UNAM), Mexico, to compare the experimental group (patients with obesity) and the control group (normal BMI patients) using the H35K chip (UNAM, Mexico) containing 35,764 genes of the human genome. The lists of DEGs (Z score ≥ 1.5 SD) in patients with obesity respect control group were obtained and analyzed as previously described [17–19] using the DAVID Bioinformatics Resources 6.8 (https://david.ncifcrf.gov/) [20], STRING 11.5 database (https://string-db.org/) [21], Cytoscape software [22] v. 3.8.2 and the Molecular Complex Detection (MCODE) plugin [23, 24], and REACTOME v83 database (https://reactome.org) [25, 26].

### 2.6. Histological Analysis

VAT biopsies from ten obese patients and ten nonobese controls were fixed in 10% buffered formalin, dehydrated in ethanol, and embedded in paraffin. Sections of 3 *μ*m were obtained and then stained with hematoxylin and eosin (H&E) for histological evaluation. The images were analyzed using a MOticEasyOne scanner (20x resolution, 0.52 *μ*m/pixel) by dividing the fields with a rack to an area of 14 mm^2^ to obtain the cross-sectional diameter of adipocytes (*μ*M), making at least 20 measurements per histological section corresponding to each patient in each study group. The means of adipocyte sizes in the VAT were subsequently compared between groups.

Immunohistochemistry (IHC) was performed with specific antibodies against tumor necrosis factor (TNF)-*α* (sc-52746), mitogen-activated protein kinase (MAPK)1 (sc-81492), and AKT serine/threonine kinase (sc-375457) (Santa Cruz Biotechnology, USA). Tissue sections were deparaffinized in two changes of xylene and dehydrated in descending concentrations of ethanol until water only. Antigen retrieval was done using 0.05% trypsin (T1426-250 mg, SIGMA Life Science, USA) and then treated with 0.2% Triton-X100 (Bio-Rad, Hercules, CA, USA). After blocking with bovine serum albumin (BSA) (A9647-100G SIGMA Life Science, USA), the tissues were incubated with the primary antibody and then with the corresponding isotype's biotin-streptavidin-conjugated secondary antibodies (Jackson ImmunoResearch Laboratories, Inc, PA, USA). Immunodetection was performed using streptavidin-HRP (Jackson ImmunoResearch Laboratories, Inc, PA, USA), with Diaminobenzidine (D4293-50SET, Sigma-Aldrich, USA) as the chromogen. The primary antibody was replaced with PBS buffer to establish a negative control. Images were acquired using a digital camera (AmScope MU1803, USA) and an optical microscope (AxioStar Plus, Carl Zeiss). The quantification of the expression of each marker was carried out with the ImageJ program and the IHC toolbox, which allowed for obtaining the ODs and their subsequent statistical analysis.

### 2.7. Statistical Analysis

The bioinformatics analysis of the data derived from DNA microarrays included statistical analysis. For OD measures in IHC, statistical analysis was made using SPSS statistics v22 software (SPSS Science Inc., Chicago, IL, USA). The Shapiro–Wilk and Kolmogorov–Smirnov tests were used to determine the data normality. In addition, means and SD were estimated and graphed, and Student's *t*-test was used to compare the expression of TNF between experimental and control groups. Differences were considered significant when *p* ≤ 0.05.

### 2.8. Ethical Aspects

The study protocol was approved by the Research Ethics Committee of the Faculty of Medicine of the Autonomous University of Chihuahua, Chihuahua, Chih., Mexico (Registry number: CI-046-20). All participants signed informed consent.

## 3. Results

The study included 10 women with obesity (36.80 ± 7.39 years) and 10 without obesity (32.70 ± 9.45 years) who were not different in age and height. The anthropometric characteristics of patients with obesity are shown in Table 1.

The microarray analysis showed that in the VAT of patients with obesity, a differential expression of 3808 genes was found (Z score > 1.5 SD): 1828 were overexpressed, and 1980 were underexpressed, regarding patients with normal BMI (Suppl. 1). According to bioinformatics analysis on the DAVID platform, DEGs in the VAT of patients with obesity were significantly associated (*p* < 0.05) with 27 biological processes and 6 KEGG signaling pathways. In comparison, those underexpressed were associated with 47 biological processes and 22 KEGG pathways (Table 2).

**Figure 1:**
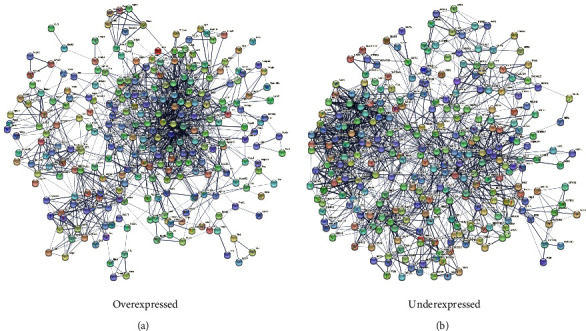
Protein–protein interaction network of differentially expressed genes in the visceral fat tissue of Mexican women with obesity (*n* = 10). The lists of the differentially overexpressed (a) and underexpressed (b) genes (Z score ≥ 1.5 SD) were analyzed on the STRING database and Cytoscape software to obtain the primary clusters of subnetworks using the Molecular Complex Detection (MCODE) complement (cutoff = 0.5). The genes from the first three clusters were loaded into the STRING database to obtain the protein–protein interaction network.

In the STRING platform, the IPP networks of the DEGs were obtained. Further analysis of these networks in the Cytoscape-MCODE clustered the over and underexpressed genes into clusters of highly interconnected regions (Figure 1). For the overexpressed genes, cluster 1 was associated with ribosome and proteasome process pathways, cluster 2 was associated with inflammatory and lipid metabolism pathways, and cluster 3 was related to essential lipid and carbohydrate metabolism pathways. For the underexpressed genes, cluster 1 was associated with ribosomal process pathways and cardiac muscle processes, and cluster 2 had several inflammatory pathways.

**Figure 2:**
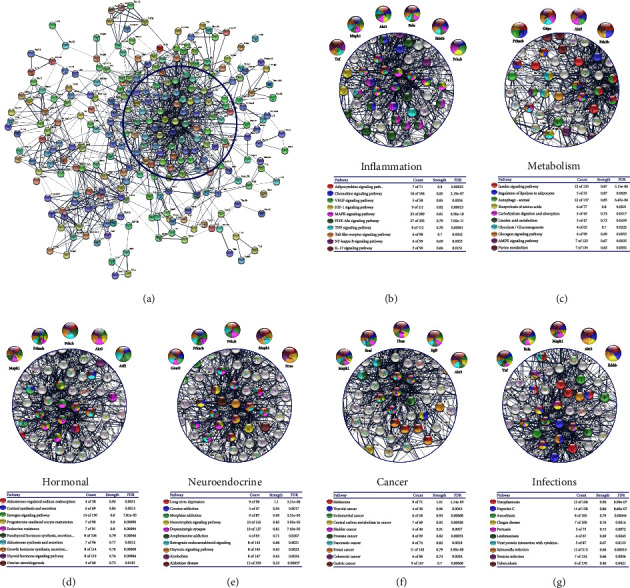
Protein–protein interaction network of differentially overexpressed genes in the visceral adipose tissue of Mexican women with obesity (*n* = 10). The lists of the differentially overexpressed genes (Z score ≥ 1.5 SD) were analyzed on the STRING database and Cytoscape software to obtain the primary clusters of subnetworks using the Molecular Complex Detection (MCODE) complement (cutoff = 0.5). The genes from the first three clusters were loaded together in the STRING database to obtain the protein–protein interaction network. In the resulting PPI network (a), the KEGG pathways (tables) related to inflammation (b), metabolism (c), hormonal (d), neuroendocrine (e), cancer (f), and infection (g) processes were marked with different colors to identify those proteins with the most significant interaction (amplified nodes).

The detailed analysis of the signaling pathways overexpressed in the VAT of patients with obesity was done in the STRING platform, where the bioinformatic analysis was subdivided into the following categories of interest in the pathogenesis of obesity: inflammation, metabolism, hormonal, neuroendocrine, cancer, and infections (Figure 2).

The overexpressed signaling pathways associated with inflammation (Figure 2(b)) included the adipocytokine, chemokine, VEGF, HIF-1, MAPK, PI3K-Akt, TNF, TLR, NF-kappa B, and IL-17 signaling pathways. The genes interacting more in those pathways were Rnf, Mapk1, Akt3, Rela, Ikbkb, and Prkcb.

Several pathways of interest in glucose and lipid metabolism were overexpressed in the VAT of patients with obesity (Figure 2(c)). These included insulin, regulation of lipolysis in adipocytes, autophagy, carbohydrate digestion, and absorption, glycolysis/gluconeogenesis, and glucose. The genes with the most significant interaction in these pathways were Akt3, Prkacb, G6pc, and Pde3b.

Hormonal signaling pathways were also significantly associated with overexpressed genes (Figure 2(d)). These included aldosterone-regulated sodium reabsorption, cortisol synthesis and secretion, estrogen, progesterone-mediated oocyte maturation, endocrine resistance, growth hormone synthesis, and ovarian steroidogenesis. The genes with higher interaction in those pathways included Mapk1, Prkacb, Prkcb, Akt3, and Atf2.

Interestingly, in the IPP resulting from overexpressed genes in VAT of patients with obesity, a lot of signaling pathways associated with addictions and neuroendocrine processes were found (Figure 2(e)). These included cocaine addiction, morphine addiction, amphetamine addiction, retrograde endocannabinoids, and alcoholism, as well as neurotrophin, dopaminergic synapse, long-term depression, and Alzheimer's disease. The more relevant genes in these pathways were Gnai2, Prkacb, Prkcb, Mapk1, and Hras.

As shown in Figure 2(f), the overexpressed genes in VAT of patients with obesity were also associated with cancer-related pathways, including melanoma, thyroid, endometrial, bladder, breast, and gastric. The genes more relevant in these pathways were Mapk1, Braf, Hras, Egfr, and Akt3.

**Figure 3:**
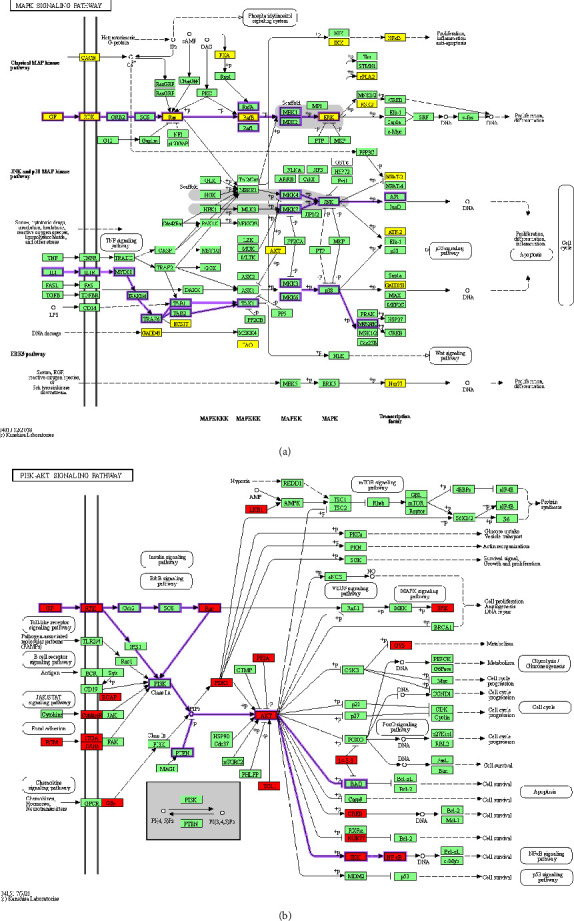
Overexpression of MAPK and PI3K-AKT signaling pathways in the visceral adipose tissue of Mexican women with obesity. Overexpressed genes significantly associated with MAPK (a) and PI3K-AKT (b) signaling pathways are shown in yellow and red, respectively. In the MAPK pathway, purple arrows indicate the deregulation of ERK and IL1-IL1R-JNK/P38 signaling, while in the PI3K/AKT pathway, they indicate the growth factors and extracellular matrix receptors' leading pathways. *n* = 10 per group.

In the IPP networks, the presence of Akt3 and Mapk1 mediators as crossover points among pathogenic pathways was consistent (Figure 2), suggesting the crucial role of PI3K/AKT and MAPK signaling pathways commanding the complex pathogenesis of obesity. A more detailed analysis of DEGs in these pathways was done in the KEGG database (Figure 3). In the PI3K/AKT pathways, DEGs were associated with the growth factors and extracellular matrix receptors leading pathways. For the MAPK pathway, the deregulation of ERK and IL1-IL1R-JNK/P38 signaling was found.

**Figure 4:**
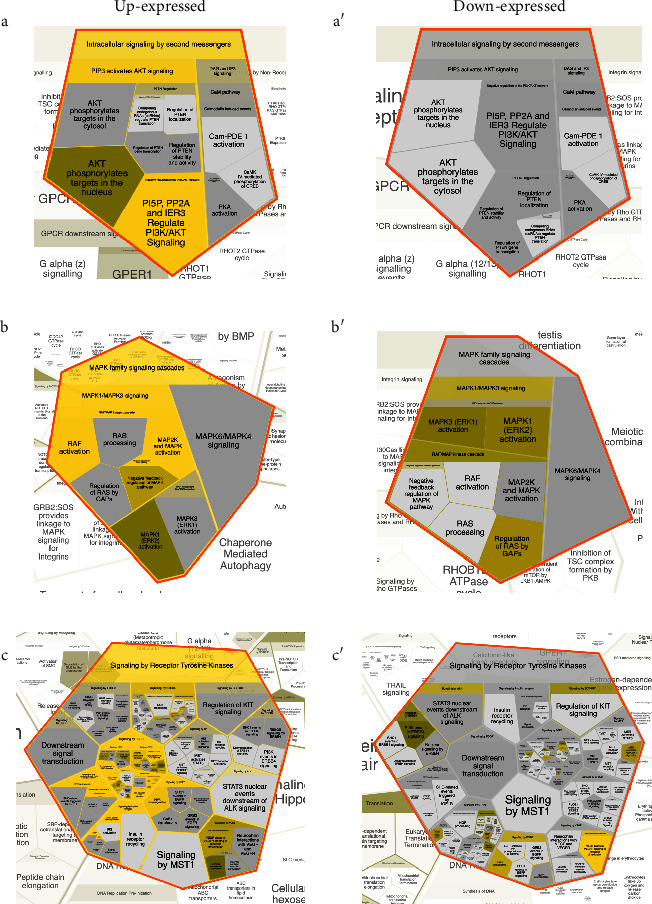
Voronoi visualization of differentially over and underexpressed genes in the visceral adipose tissue of Mexican women with obesity. The lists of the differentially over and underexpressed genes (Z score ≥ 1.5 SD) were analyzed on the REACTOME database to obtain Voronoi visualization. Amplification of the intracellular signaling by second messengers (a and a′), MAPK family signaling cascades (b and b′), and signaling by receptor tyrosine kinases (c and c′) was done for a deeper visualization. *n* = 10 per group.

The analysis in the REACTOME database generated the Voronoi visualization, which confirmed the association of overexpressed (Suppl. 2) and underexpressed genes (Suppl. 3) with pathways of the immune system, including inflammation, metabolism, cell cycle, and signal transduction. A deeper analysis of REACTOME allowed the visualization of the association of over and underexpressed genes in more specific processes, in which the associations of overexpressed genes were overwhelming. Interestingly, the processes of the neuronal system were practically only overexpressed. Moreover, as found in STRING and Cytoscape analysis, the overexpression of intracellular mediators and receptors of PI3K/AKT, MAPK, and receptor tyrosine kinase signaling was confirmed in REACTOME (Figure 4).

**Figure 5:**
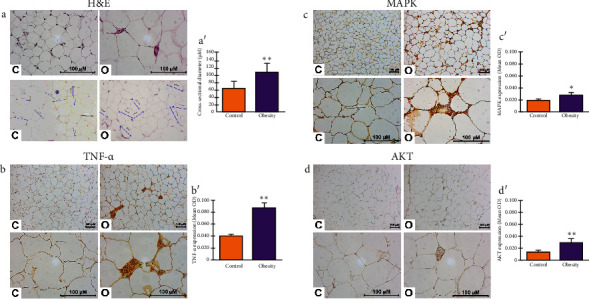
Overexpression of TNF-*α*, MAPK, and AKT proteins in the visceral adipose tissue of Mexican women with obesity. Representative images of histological analysis evaluated in H&E staining (a) and quantitative evaluation (a′) of the adipocytes' cross-sectional diameter of VAT of women with obesity (O) and control (C) women. Representative images of immunodetection of TNF-*α* (b), MAPK (c), and AKT (d) in VAT of women with obesity (O) and control (C). The images were acquired with 10X and 40X amplification. Optical density (OD) means and standard deviations are shown in graphics (b′, c′, and d′). Student's *t*-test was used to determine statistically significant differences. ⁣^∗^: *p* ≤ 0.05; ⁣^∗∗^*p* ≤ 0.01. *n* = 10 per group.

The histological analysis showed that the adipocyte size of patients with obesity was significantly larger in diameter than those of the control group (Figures 5(a) and 5(a′)). Moreover, the overexpression of TNF-*α* (Figures 5(b) and 5(b′)), MAPK (Figures 5(c) and 5(c′)), and AKT (Figures 5(d) and 5(d′)) was demonstrated in the VAT of patients with obesity compared to patients without obesity through IHC.

## 4. Discussion

The present study compared the transcriptome profiles of VAT of Mexican women with and without obesity and confirmed the deregulation of several signaling pathways related to metabolism, inflammation, cancer, infections, hormonal processes, and addictive behaviors. The interconnection of these pathways through Mapk1 and Akt3 in the VAT of patients with obesity was also demonstrated; to our knowledge, this is the first peer-reviewed report of a transcriptomic analysis that establishes these molecules as crossing points in the complex pathogenesis of obesity. The overexpression of TNF-*α*, MAPK, and AKT protein was confirmed in the VAT of patients with obesity.

Previous studies have described the VAT transcriptomic profile in different populations, only one in the Mexican population [27]. Most of them, including the study in Mexico, focused on comparing the VAT and SAT transcriptome of patients with obesity [27–29], and the minority shared the experimental design with us and compared the VAT expression profiles of patients with obesity with those of normal weight [30]. The VAT and SAT transcriptomes have been confirmed to be different, identifying genes (Il6, Il8, Il18, Saa1, Ccr2, Mif, Mvd, Acsl3, Sucgl, Idh2, Ppap2c, Cyp4a11, Cyp1b1, Pgc1-Α, and Fncd5) and signaling pathways (inflammation, oxidative stress, insulin homeostasis, and lipid metabolism) that are differentially expressed between these tissues [28, 31, 32]. On the other hand, when comparing the VAT of patients with obesity with that of normal weight donors, genes and pathways related to glucose, lipid and amino acid metabolism, membrane transport, cell cycle, and inflammation [14, 30] have been identified. These findings are consistent with ours; however, our bioinformatics analysis was extended to identify crossing points between the pathways deregulated in VAT in patients with obesity.

Our study finely details the VAT transcriptomic profile of patients with obesity, showing the dysregulation of inflammatory pathways, many of which relate to innate immune response and Toll-like receptor, NF*κ*B, and TNF, which are physiologically connected. Several intracellular kinases, including those of MAPK and PI3K-AKT pathways, were also overexpressed. Rela, which was also found to be overexpressed, is indeed a part of Nf-kB and is connected to TNF [33] and TLR [34]. The proinflammatory profile of VAT in obesity has undoubtedly been demonstrated in previous studies analyzing the transcriptome [14, 30].

Regarding metabolism, as expected, several pathways related to carbohydrate and lipid metabolism show dysregulation in obese patients' VAT. The main genetic cross points behind these pathways include Akt3 and other regulatory genes like phosphodiesterase 3b (Pde3b), expressed on adipocytes and linked to the regulation of the inflammasome. Pde3b is involved in insulin signalization and is related to obesity and diabetes [35]. Also, glucose-6-phosphatase (G6pc) is mainly in non-gluconeogenic tissues and is critical in carbohydrate metabolism [36].

Additionally, we found dysregulation in pathways related to neuroendocrine processes, including those linked to addiction (to cocaine, morphine, amphetamine, and alcohol). Interestingly, VAT participated in this behavioral profile. Sugar addiction [37] is recognized as an additional complicating factor in the battle against obesity and, like addiction to other substances, profoundly influences subconscious eating behavior [38, 39]. Mapk1, Prkacb, and Prkcb were also identified as crossing points among several pathways. Gnai2 (coding for G Protein Subunit alpha I2) was also overexpressed in our study. Mice with deficient Gnai2 signaling do not develop obesity despite a high-fat diet and show enhanced insulin sensitivity [40]. G-protein regulators are relevant in addiction-related behaviors [41].

Obesity is a condition predisposing to various cancers [42]. In the VAT, we found a dysregulation in signaling pathways linked to multiple cancers, predominantly glandular cancers of the digestive and genitourinary tracts; also, the pathways' cross points included Akt3 and Mapk1. Additionally, the Hras and Braf genes, which are linked to oncogenesis [43, 44] and the latter considered a therapeutic target [45], were overexpressed in VAT of patients with obesity in our study.

We found that Akt3 is a cross point in the PPI networks deregulated in the VAT of patients with obesity. Akt3 belongs to the PI3K/AKT signaling pathway; it regulates several critical cellular processes, including proliferation, differentiation, apoptosis, cell size, and response to nutrient availability, tumorigenesis, and insulin and growth factors. AKT signaling pathway has gained research interest since its relation to a great variety of cancers [46, 47] but also seems linked to the pathogenesis of epilepsy [48], osteoporosis [49], and several more.

PI3K/AKT is the major effector of insulin metabolic actions [50]. The PI3K/AKT signaling pathway is highly dependent on the signaling context and the specific effector involved (AKT1, AKT2, or AKT3). Although the roles of AKT1/2 in adipogenesis and insulin signaling are defined, the role of AKT3 in adipocytes has been sparsely assessed. Mice lacking AKT3 have enhanced adipogenesis via serum/glucocorticoid-inducible kinase 1 (SGK-1) [51]. As can be inferred, AKT defines the consequential cell behavior under a diversity of stimuli, or furthermore, under specific environments as the result of constant multiple stimuli, such as obesity, which is defined by a perennial excess of nutrients, persistently triggering downstream pathways linked to the presence of specific hormones like insulin or highly available intracellular nutrients. PI3K/AKT pathway additionally connects to the mammalian target of rapamycin (mTOR), which sets an anabolic profile in the cells, increasing protein synthesis and cell division and blocking autophagy [52]. Therefore, persistent mTOR activity has been connected to aging [53], inflammation [54], and carcinogenesis [55]; all those conditions have also been linked to obesity. As we show in our results, VAT directly plays a role in setting this profile.

MAPK1 is also a cross point in several PPI networks in our analysis. MAPK1 (ERK2) is a serine/threonine kinase that transduces and integrates several biochemical signals and induces several cellular behaviors such as proliferation, differentiation, and development. MAPK1 has been linked to leptin-resistant obesity and obesity-related precocious puberty and is considered a potential therapeutic target for obesity [56]. The connection between AKT and MAPK1 has been shown since both signals are a mechanism for insulin resistance in adipocytes through the nuclear factor Elk1 [57]. The differentiation of mesenchymal stem cells into adipocytes is associated with the PI3K/AKT and MAPK1 signaling pathways using insulin as the priming stimulus. It has been shown that senescent mesenchymal stem cells exhibit high basal levels of phosphorylation in both ERK1/2 and AKT, regardless of the presence of insulin [58].

Although PI3K/AKT and MAPK1 signaling pathways and their Akt and Mapk mediators have been associated with the pathogenesis of obesity, their role as crossing points between the signaling pathways, which typically are studied in isolation, has not been previously described. The description of these linking mechanisms between the most relevant processes of obesity, including inflammation, metabolism, and addictive behavior, could lead to more comprehensive diagnoses and treatments.

This study has limitations that deserve to be mentioned. The observational design of our study restricts the interpretation of our results, providing us with descriptive results; however, the findings contribute to the current understanding of obesity at the transcriptomic level. Furthermore, despite our best efforts, the number of patients we included in our analysis constituted a small sample; nevertheless, we believe that the robustness of the transcriptome analysis allows us to drive conclusions given the substantial number of internal quantifications and the fact that the comparisons reached statistical significance. Finally, we recognize that the control (nonobese) group lacks several descriptors related to the anthropometry and we are limited to general demographics and BMI; as was mentioned, the control group had a BMI < 25 kg/M^2^, therefore not considered to be obese. Obtaining VAT samples from healthy individuals presents ethical challenges. We did not have complete access to the control group patients scheduled for surgery who had consented to provide samples. Nonetheless, we acknowledge the limited availability of anthropometric data. As mentioned, the BMI data (22.5 kg/m^2^ vs. 44.21 kg/m^2^) allow an undoubted separation of the groups, later confirmed by the transcriptome. On the other hand, given the high prevalence of obesity among Mexican women, incorporating nonobese people into the control group turned out to be a more significant challenge, in addition to the fact that we never considered including healthy patients who were not scheduled for elective surgery and subject them to VAT sampling. Furthermore, we could not completely rule out the potential influence of an inflammatory process related to the gynecological condition in the control group. However, if such inflammation had a significant impact, it would likely have reduced the differences between the study groups. Unfortunately, healthy donors for VAT samples were not available.

## 5. Conclusions

VAT plays a preeminent role in the complex picture of obesity because its transcriptomic profile replicates many of its critical pathogenic consequences. These key pathogenic processes rely on intracellular signaling chains that can be primed and persistently activated by the abnormal metabolic environment, fundamentally the constant glucose load and persistent insulin signaling throughout its receptor. We found that the overexpression of Akt3 and Mapk1 is a constant crossroad for inflammation, metabolic abnormalities, addictive behaviors, and cancer signaling pathways, suggesting a notable role. However, that opens the possibility that the controlled modulation of either or both would significantly impact downplaying these pathogenic processes; if feasible, many current burdens bound to obesity could be diminished.

## Figures and Tables

**Table 1:** Anthropometric characteristics of the cases and control groups.

Variable	Normal weight Mean ± SD (*n* = 10)	Obesity Mean ± SD (*n* = 10)	*p* a
Age (years)	32.70 ± 9.45	36.80 ± 7.39	0.294
Height (cm)	161 ± 4.19	163 ± 6.62	0.429
Weight (kg)	58.45 ± 7.64	117.68 ± 19.11	<0.001
BMI (kg/m^2^)	22.52 ± 2.54	44.21 ± 6.01	<0.001

**Anthropometric characteristics of patients with obesity**			
**Variable**		**Mean ± SD (*n* = 10)**	

% fat		50.24 ± 5.54	
Body fat (kg)		59.61 ± 14.37	
Lean weight		58.08 ± 6.12	
Visceral fat scale		15.77 ± 3.13	
% water		37.14 ± 4.25	
Water weight (kg)		43.29 ± 5.39	
Muscle weight (kg)		55.14 ± 5.83	
Basal metabolic rate (Kcal)		1836.10 ± 212.45	
Metabolic age		89.40 ± 1.89	
Daily caloric intake		2803.60 ± 537.35	

^a^ *t*-test.

**Table 2:** KEGG signaling pathways associated with the differentially expressed genes in visceral adipose tissue from patients with obesity.

**Overexpressed**	**Underexpressed**
**KEGG pathway** (count; *p* value): *genes*	**KEGG pathway** (count; *p* value): *genes*

**Long-term depression** (12; 1.00e-03): *Braf, Gnai2, Hras, Crhr1, Gria3, Mapk1, Mapk3, Pla2g4c, Plcb2, Prkg1, Ppp2r1a, Ryr1* **Glutamatergic synapse** (17; 1.80e-03): *Gnai2, Gnb4, Gng13, Shank3, Adcy5, Dlg4, Gria3, Grin2d, Grm2, Mapk1, Mapk3, Pla2g4c, Plcb2, Prkacb, Slc1a1, Slc1a7, Slc17a6* **Insulin signaling pathway** (18; 5.20e-03): *Pdpk1, Akt3, Braf, Cbl, Hras, Rapgef1, G6pc, Gys1, Ikbkb, Lipe, Mapk1, Mapk3, Pde3b, Prkacb, Prkar2a, Sorbs1, Socs4, Trip10* *T Cell Receptor Signaling Pathway (14; 8.90e-03): Pdpk1, Akt3, Cd28, Cd40lg, Cd8b, Hras, Rela, Csf2, Ikbkb, Mapk1, Mapk3, Nfatc1, Ptprc, Vav3* **Cocaine addiction** (9; 1.00e-02): *Gnai2, Rela, Atf2, Adcy5, Cdk5r1, Dlg4, Grin2d, Grm2, Prkacb* **Circadian entrainment** (13; 1.50e-02): *Gnai2, Gnb4, Gng13, Adcy5, Gria3, Grin2d, Mapk1, Mapk3, Plcb2, Prkacb, Prkg1, Ryr1, Ryr3* **Pi3k-Akt signaling pathway** (33; 1.50e-02): *Pdpk1, Akt3, Gnb4, Gng13, Hras, Rela, Tcl1b, Atf2, Egfr, Fgf1, Fgf19, Fgf6, Fgf9, Flt1, Flt4, G6pc, Gys1, Ikbkb, Ibsp, Itga5, Itga6, Itgav, Itgb6, Mapk1, Mapk3, Nr4a1, Osmr, Pik3ap1, Ppp2r5b, Ppp2r1a, Stk11, Thbs2, Ywhae* **Shigellosis (**10; 1.70e-02): *Rela, Cttn, Diaph1, Elmo1, Ikbkb, Itga5, Mapk1, Mapk3, Pfn4, Vcl* **Pertussis** (11; 1.70e-02): *Gnai2, Rela, Casp1, Cfl2, C1s, C5, C4bpa, Itga5, Mapk1, Mapk3, Ticam2* **Hepatitis C** (16; 1.80e-02): *Pdpk1, Akt3, Braf, Hras, Rela, Cldn1, Cldn10, Cldn17, Egfr, Eif2ak4, Ikbkb, Ldlr, Mapk1, Mapk3, Ppp2r1a, Tyk2* **Serotonergic synapse (**14; 2.00e-02): *Htr2a, Htr4, Braf, Gnai2, Gnb4, Gng13, Hras, Adcy5, Alox15, Mapk1, Mapk3, Pla2g4c, Plcb2, Prkacb* **Cholinergic synapse** (14; 2.00e-02): *Akt3, Gnai2, Gnb4, Gng13, Hras, Adcy5, Chat, Chrna3, Mapk1, Mapk3, Plcb2, Kcnq2, Kcnq4, Prkacb* **Regulation of actin cytoskeleton** (22; 2.10e-02): *Braf, Fgd1, Git1, Hras, Cfl2, Diaph1, Egfr, Fgf1, Fgf19, Fgf6, Fgf9, Itga5, Itga6, Itgad, Itgav, Itgb6, Mapk1, Mapk3, Myl2, Pfn4, Vav3, Vcl* **Ras signaling pathway** (23; 2.40e-02): *Akt3, Gnb4, Gng13, Hras, Rela, Egfr, Fgf1, Fgf19, Fgf6, Fgf9, Flt1, Flt4, Ikbkb, Ksr1, Ksr2, Mapk1, Mapk3, Pla1a, Pla2g4c, Pla2g5, Pla2g6, Prkacb, Syngap1 ***mTOR signaling pathway** (9; 2.60e-02): *Pdpk1, Akt3, Braf, Cab39, Ikbkb, Mapk1, Mapk3, Rps6ka2, Stk11* **B cell receptor signaling pathway** (10; 2.70e-02): *Akt3, Btk, Hras, Rela, Ikbkb, Mapk1, Mapk3, Nfatc1, Pik3ap1, Vav3* **Melanoma** (10; 3.10e-02): *Akt3, Braf, Hras, Egfr, Fgf1, Fgf19, Fgf6, Fgf9, Mapk1, Mapk3* **Chronic myeloid leukemia** (10; 3.40e-02): *Akt3, Braf, Bcr, Ctbp2, Cbl, Hras, Rela, Ikbkb, Mapk1, Mapk3* **MAPK signaling pathway** (24; 4.30e-02): *Akt3, Braf, Ddit3, Ecsit, Hras, Rela, Taok2, Atf2, Cacng7, Egfr, Fgf1, Fgf19, Fgf6, Fgf9, Gadd45g, Ikbkb, Mapk1, Mapk3, Ntf3, Nfatc1, Nr4a1, Pla2g4c, Prkacb, Rps6ka2* **Estrogen signaling pathway** (12; 4.30e-02): *Akt3, Fkbp5, Gnai2, Hras, Atf2, Adcy5, Egfr, Mapk1, Mapk3, Oprm1, Plcb2, Prkacb* **Gap junction** (11; 4.60e-02): *Htr2a, Gnai2, Hras, Adcy5, Egfr, Mapk1, Mapk3, Plcb2, Prkacb, Prkg1, Tjp1* **Retrograde endocannabinoid signaling** (12; 4.90e-02): *Gnai2, Gnb4, Gng13, Abhd6, Adcy5, Gabra1, Gria3, Mapk1, Mapk3, Plcb2, Prkacb, Slc17a6*	**HTLV-I infection** (25; 3.60e-03): *Apc, Bub1b, Crtc3, Pold1, Pole4, E2f3, Elk4, Gps2, Jak1, Tnfrsf1a, Wnt10b, Wnt5b, Wnt6, Wnt8a, Atf3, Adcy6, Anapc10, Cdc26, Ccnd1, Dlg1, Dvl3, Map3k3, Pik3ca, Pik3r1, Slc25a5* **Platelet activation** (14; 1.80e-02): *Gnaq, Adcy6, F2r, Col3a1, Fgb, Fgg, Gp1ba, Gucy1a2, Gucy1a3, Pik3ca, Pik3r1, Plcb1, Ppp1r12a, Tbxas1* **Jak-STAT signaling pathway** (15; 2.00e-02): *Jak1, Mpl, Csf2ra, Ccnd1, Ifna16, Il12rb1, Il12a, Il22ra2, Il23r, Il7r, Pik3ca, Pik3r1, Pias4, Ptpn11, Socs5* **Hippo signaling pathway** (15; 2.70e-02): *Apc, Wnt10b, Wnt5b, Wnt6, Wnt8a, Bmp5, Bmpr1b, Crb1, Ccnd1, Dlg1, Dvl3, Nf2, Pard3, Stk3, Ywhaz* **Cardiac muscle contraction (**9; 4.20e-02): *Fxyd2, Cacna1c, Cox6b2, Cox7b2, Myh6, Myl3, Tpm1, Tpm3, Tnnc1* **Chagas disease** (American Trypanosomiasis) (11; 4.60e-02): *Ccl3l3, Faslg, Gnao1, Gnaq, Traf6, Tnfrsf1a, Irak1, Il12a, Pik3ca, Pik3r1, Plcb1*

## Data Availability

The data that support the findings of this study are available on request from the corresponding authors. The data are not publicly available due to privacy or ethical restrictions.

## Supporting Information

Supporting Information Supplementary 1. List of differentially expressed genes in VAT of women with obesity compared with nonobese controls resulted from microarray analysis.

Supporting Information Supplementary 2. Voronoi visualization of overexpressed genes in VAT of women with obesity obtained in the REACTOME v83 database.

Supporting Information Supplementary 3. Voronoi visualization of underexpressed genes in VAT of women with obesity obtained in the REACTOME v83 database.
