# Supplementary figures and images for: Microarray Analysis of Visceral Adipose Tissue in Obese Women Reveals Common Crossroads Among Inflammation, Metabolism, Addictive Behaviors, and Cancer: AKT3 and MAPK1 Cross Point in Obesity
Source: J Obes. 2024 Oct 24;2024:4541071. doi: 10.1155/2024/4541071 (PMC11527533; doi:10.1155/2024/4541071)

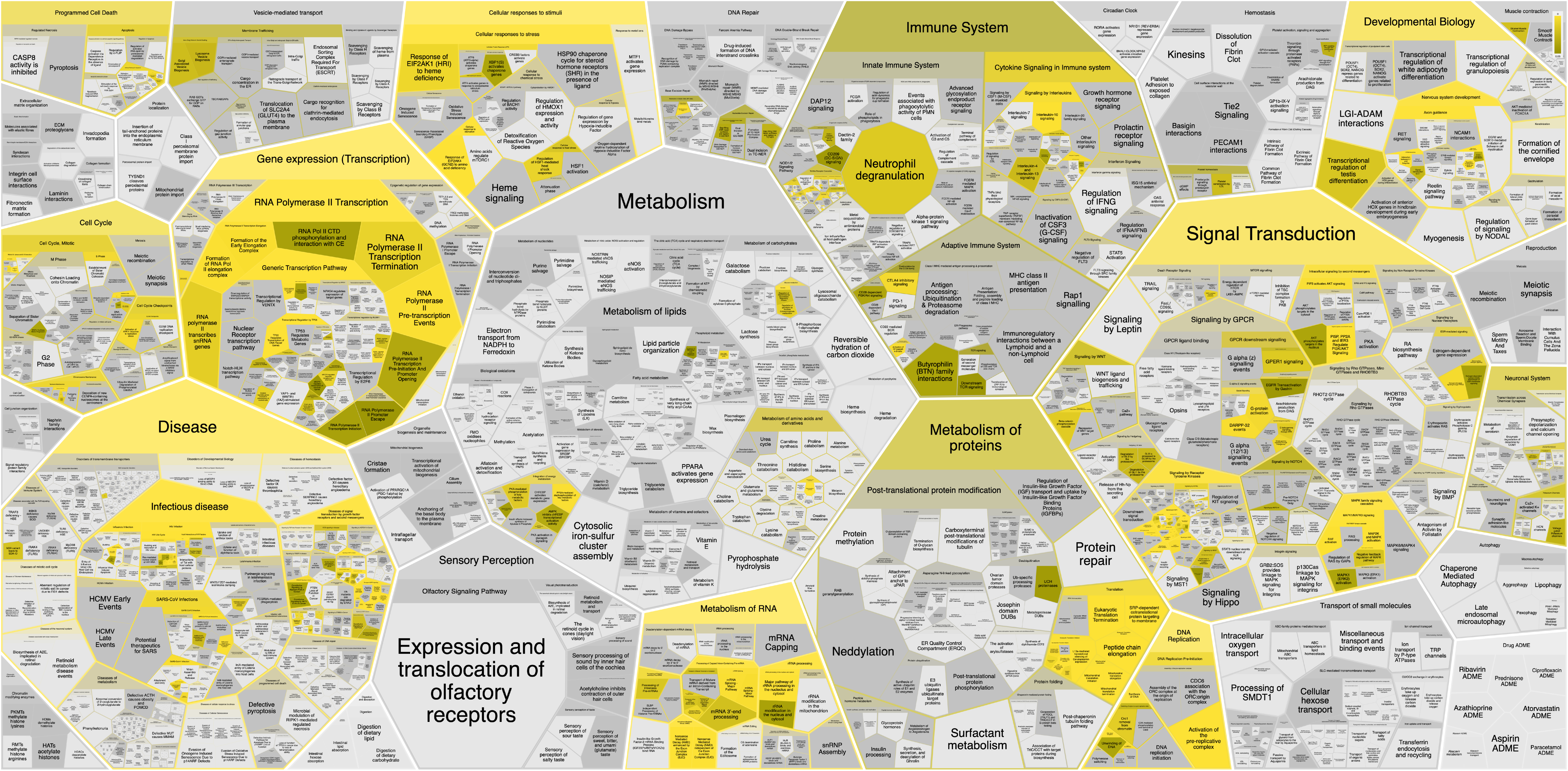

Supplement: Supporting Information — Supplementary 2. Voronoi visualization of overexpressed genes in VAT of women with obesity obtained in the REACTOME v83 database. [file 4541071.f2.pdf]

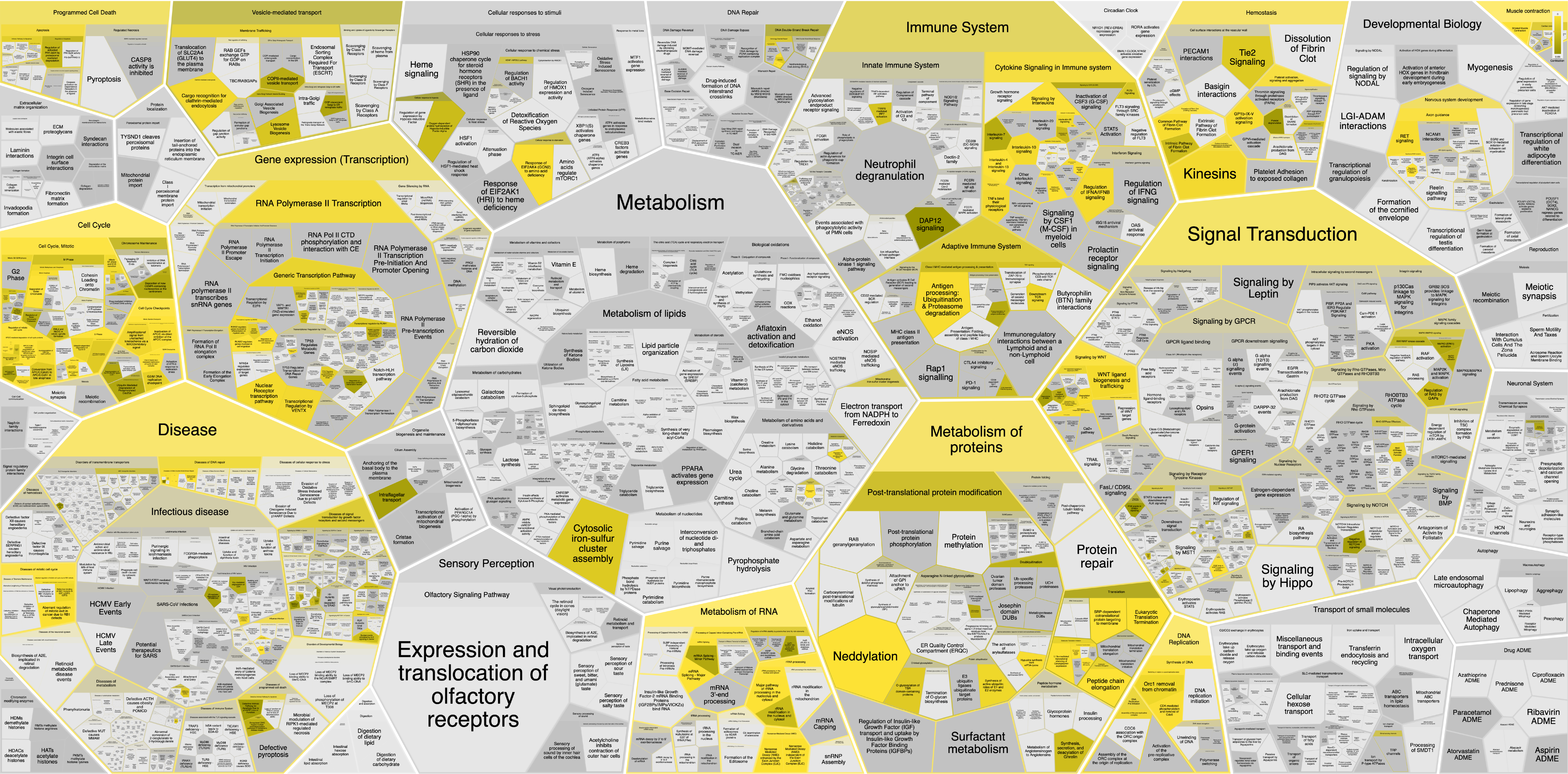

Supplement: Supporting Information — Supplementary 3. Voronoi visualization of underexpressed genes in VAT of women with obesity obtained in the REACTOME v83 database. [file 4541071.f3.pdf]
